# Life history traits in a capital breeding pine caterpillar: effect of host species and needle age

**DOI:** 10.1186/s12898-018-0181-0

**Published:** 2018-08-08

**Authors:** Dan Luo, Meng Lai, Chuanfeng Xu, Haoni Shi, Xingping Liu

**Affiliations:** College of Forestry, Jiangxi Agricultural University, Nanchang, 330045 Jiangxi China

**Keywords:** Host species, Needle age, Larval performance, Mating success, Fitness consequences, Nutritional quality

## Abstract

**Background:**

For capital breeding Lepidoptera, larval food quality is a key determinant of their fitness. A series of studies have suggested that the larval host species or varieties dramatically impact their development and reproductive output. However, few studies have reported the role of foliar age and adult mating success has often been ignored in these studies. In this paper, the influence of host species and needle age on larval performances, adult mating behavior and fitness consequences has been studied using a capital breeding caterpillar, *Dendrolimus punctatus* Walker (Lepidoptera:Lasiocampidae).

**Results:**

In larval performance trial, a strong effect of larval host species and needle age was found on survivorship, developmental duration, body weight, percentage of adult emergence, and growth index, but not on percentage of female progeny. In adult mating trial, larval host species and needle age also significantly affected mating latency and mating duration, but not mating success. In adult fitness trial, female fecundity, longevity and fitness index, but not oviposition duration and fertility, influenced by larval host species and needle age.

**Conclusions:**

These results reveal the importance of larval host species and needle age on larval performance and adult reproductive fitness in this capital breeding insect and provide strong evidence that old needles of masson pine *P. massoniana* is the best host for *D. punctatus*.

## Background

Interactions of herbivorous insects with their host plants play important roles in the evolution of a variety of traits. Herbivorous insects can use diverse feeding strategies to obtain required nutrients from their host plants to ensure their development and reproduction [1]. Numerous experimental studies have been performed to examine the influence of host plants quality on their life history traits in recent decades. All of these studies have confirmed that plant quality dramatically impact larval development and adult reproductive output [2]. Capital breeding, as one of an important reproductive strategy, is common in many Lepidoptera. Their reproductive potential critically depends upon resource accumulation during their larval stage [3]. Therefore, larval host plants quality is a key determinant of their fitness for their population increases and outbreaks [2–6]. In general, high quality food ingested by larvae promotes larval performance, as a consequence, enhances adult reproductive output [2, 4–12].

The variance in both larval performance and reproductive output of herbivorous insects is often attributed in large part to the nutritional quality of host plants. Indeed, the nutritional quality of host plants varies naturally among different species [6, 7, 9, 11, 13–15], or different varieties within cultivated species [16, 17]. In some conifers, however, host nutritional quality usually changes as the growing season progresses [18–21]. In this case, it is expected that life history characteristics of conifer-feeding insects may be influenced by foliar age [22]. To the best of our knowledge, only few studies have reported that herbivorous insects can enhance their performance by feeding on particular age classes of foliage from a single host plant species [16, 23–25]. Meanwhile, most current studies take into account only a few fitness parameters, female mating success has often been ignored, even though this parameter seems crucial in determining the fitness of herbivorous insects [2]. To better assess the quality and suitability of host plants and explore the adaptation mechanisms of insects, more relevant studies are needed.

The pine caterpillar, *Dendrolimus punctatus* Walker (Lepidoptera:Lasiocampidae), is the most destructive pine defoliator of conifer forests, causing large economic losses and ecological effects in the south of China. This caterpillar occurs in 17 provinces and regions in China [26, 27] and in Southeast Asia [28]. This species usually produce 2–4 generations in different regions, and overwinters as third to fourth instar larvae [26, 29]. The larvae have 6–7 instars and feed gregariously on fresh pine needles [27, 30]. Mature larvae make their cocoon typically near the tip of branch or treetop. This caterpillar is considered as a capital breeder because their adults do not feed and have short lifespan [26]. Therefore it is essential that larvae receive all necessary nutrients and other substances to survive and reproduce, making the larval food critical to the fitness of the adults. Previous studies showed that the preferred host plants mainly include a native species, masson pine *Pinus massoniana* Lamb and an exotic species, slash pine *P. elliottii* Engelm [26, 29]. Further investigations have been carried out by He et al. [31], and revealed that this caterpillar suffered direct survival cost from feeding slash pine in larval stage, confirming that the role of host plants species may be much more important in larval development and survival. Despite the differences observed, no study has explored whether host plants species or needle age classes have any impact on the mating activity and reproductive success in adult stage. A comprehensive study of the impacts of host plants on *D. punctatus* reproduction will provide further insight into the complex interactions between plants and herbivorous insects. The results could also be highly useful for the better understanding of host plant resistance mechanism and for evolving successful management strategies for this pest.

The main purpose of this study was to examine the influence of larval food quality on a whole suite of life-history traits in this caterpillar. Variation in larval food quality was obtained by offering to the larvae two pine species with two needle age types respectively. We first performed larval rearing trial from egg hatching to adult emergence to find out whether pine species and needle age affect larval performances, by measuring the total developmental duration, larval and pupal survivorship, pupal body size, percentage of adult emergence, percentage of female progeny and growth index. Then we conducted adult trials from individual emergence to death to estimate the effect of larval food quality on adult mating behavior and fitness consequences. In the first part, we recorded adult mating behavior such as the proportion of mating success, mating latency and mating duration. In the last part, we measured the components of adult fitness in terms of oviposition duration, fecundity, egg hatchability, adult longevity and fitness index. The results may aid our understanding of the adaptation of *D. punctatus* to host plants.

## Methods

### Insects and plant materials

A laboratory colony was established from field collected larvae in Wan’an County, Jiangxi province, China (26°20′N, 115°0′E) in April 2015. The field collected larvae were maintained on transparent plastic boxes (20 cm × 15 cm × 7 cm) with masson pine needles as larval food and reared in artificial climate incubators. Upon maturation, the larvae were allowed to form cocoons, and pupae were harvested and placed into empty cages (30 cm × 30 cm × 40 cm). Newly emerged adults (on the same evening, within 5 h) were transferred into new cages with fresh branches of masson pine as mating and oviposition arena. Eggs produced by females were afterwards collected and transferred into plastic rearing boxes (7.5 cm × 7.5 cm × 6 cm) lined with moist filter for further development. The insects had been maintained in the laboratory for about six generations on masson pine needles. Before starting the experiments, we subdivided the laboratory population. Some of larvae were reared on masson pine needles and some on slash pine needles for whole a generation (from eggs to adult emergence) to allow them to adapt to the new host plants and to remove maternal effects. All insect rearing and experiments were conducted under the same ambient conditions at 28 ± 1 °C and 70 ± 10% RH with 14 h light: 10 h dark regime in artificial climate incubators (larvae rearing and adult fitness trials) and air-conditioned chamber (behavior observation trial).

Two pine tree species, masson pine *P. massoniana* and slash pine *P. elliottii*, were collected from local forestry near the campus of Jiangxi Agricultural University. Both of which are common in local forestry. The current-year and previous-year needles were haphazardly collected from each tree species, respectively. Therefore, four treatments in this study were used according to larval food species and needle age in our experiments, including current-year needles of *P. massoniana*, previous-year needles of *P. massoniana*, current-year needles of *P. elliottii* and previous-year needles of *P. elliottii*. All plant materials used were grown under field conditions without damaged and sprayed any pesticides. Branches of four types of larval food were collected every 3 days and were washed in 0.06% sodium hypochlorite for 5 min and then rinsed in water, shortly afterwards stored at 4 °C in a refrigerator and used for experiments.

### Larval performance trial

In order to determine the relative quality of host plants for larvae of *D. punctatus*. The neonate larvae (total *n* = 400) of *D. punctatus* were equally divided into four food treatments mentioned above. In each treatment, one neonate larva was placed individually into a transparent plastic 50-mL tube with ventilated lid. Tubes with larvae were thereafter transferred to the artificial climate incubators to allow larval development. Fresh needles were used as the larvae food sources and were replaced every day. Survival of the larvae was checked every day until they built cocoons inside the tubes. Pupae of each treatment were extracted 48 h after cocoon formation and body weights were measured using electronic balance to the nearest 0.1 mg (model FA2004, Shanghai, China). Pupae were then placed individually into new transparent plastic tubes to allow adult emergence. The tubes were checked daily and then the emerged adults were sexed. Thus, for each treatment, we quantified important larval and pupal life-history traits comprising: (i) larval and pupal survivorship; (ii) larval and pupal developmental duration; (iii) pupal body weight; (iv) percentage of adult emergence; (v) percentage of female progeny; and (vi) growth index, expressed as pupal weight (mg)/larval development time (days) [32, 33].

### Adult mating trial

To determine the influence of the larval host plants on adult mating behavior and adult fitness measurement, 30 neonate larvae of *D. punctatus* were carefully introduced into a transparent plastic box (20 cm × 15 cm × 7 cm) with a 10-cm-long branch of one of the four food sources. The boxes were then transferred to the artificial climate incubator to allow larval development. Ten replicates with a total of 300 larvae were conducted for each food source. Thirty pairs of newly emerged adults originating from each food sources were used in this trial. For each treatment, one newly emerged virgin female and one newly emerged male originating from the same larval diet were paired 4 h prior to lights off and placed in a large transparent plastic cylinder (12 cm in diameter × 18 cm in height) containing a fresh branch of corresponding larval host as mating arena. Subsequently, mating behavior was observed at 30-min intervals until the end of the scotophase or until copulation finished. Observations in scotophase were made using a flashlight at 0.3 lx covered by several pieces of red cellophane. We recorded the following variables: (i) mating latency, defined here as the time elapsed from when the adults were introduced into the mating arena to when pairs started coupling; (ii) copulation duration, assessed by the time elapsed from when coupling started to when the pairs physically separated; and (iii) proportion of mating success.

### Adult fitness trial

To estimate the influence of larval food quality on adult fitness consequences, five males and five females developed from same larval food source were pooled and transferred into a large transparent plastic cylinder containing a fresh branch of corresponding host plants as mating arena. Six replicates with a total of 30 males and 30 females were conducted for each food source. Observation was conducted at 30-min intervals. When a pair successfully copulated, the mated pair was then gently transferred from the cylinder into a small transparent plastic cylinder (5 cm in diameter × 10 cm in height) using soft forceps. A fresh branch same as larval host was supplied to each pair as mating arena. When the pair finished copulation, the mated females and males were separated and transferred individually into a new transparent plastic cylinder until death. A same fresh branch as larval host was provided and replaced per day as oviposition arena for female and rest place for male. Eggs were numbered and collected daily, and then transferred into a Petri dish (9.0 cm diameter, 1.5 cm height) lined with moistened filter paper and placed into artificial climate incubators. Petri dishes were checked daily and the number and proportion of larvae hatched were counted as measures of hatching success. Dead females were dissected and the number of eggs remained under their abdomen were counted. In this trial, five parameters were considered: (i) female oviposition duration; (ii) mean number of eggs laid and remained; (iii) egg hatching rate; (iv) longevity of mated females and males; and (v) female fitness index, calculated using the formula in number of female produced × proportion of mating success [4, 34].

### Statistical analysis

All data analyses were performed using SPSS version 19.0 for Windows (SPSS Inc., Chicago, IL, USA). Two-way ANOVA were performed using univariate procedure of General linear model (GLM) to determine the influence of host species and needle age on life history characteristics of *D. punctatus*, in which one of the parameters in larval and pupal traits (developmental duration, survivorship, pupal body weight, percentage of adult emergence, percentage of female progeny, growth index) was used as dependent variables, and host species and needle age were used as fixed factors. The data on adult mating behavior (mating latency and mating duration) and adult fitness parameters (oviposition duration, fecundity, fertility, adult longevity and fitness index) were analyzed using independent-samples T test to evaluate the effect of host species and needle age respectively. The percentages of larvae and pupae survivorship, adult emergence and egg hatching were arcsine square root transformed prior to statistical analysis. Adult mating success was analyzed using Nonparametric Pearson χ^2^ test. Data are presented as mean ± SE.

## Results

### Larval and pupal traits

The pine species and needle age significantly affected larval performance (Table 1). Overall, significantly fewer larvae survived when reared on *P. elliottii* and current-year needles compared with *P. massoniana* and previous-year needles. The percentage of surviving pupae was also significantly different between different host species and different needle age. The longest duration in larval stages was observed in the current-year needles of *P. elliottii* treatment and the shortest duration was found in the previous-year needles of *P. massoniana* treatment. The statistics showed that larval developmental duration varied significantly among pine species and needle age on which larvae were fed. A similar tendency was observed for the pupal developmental duration. The food consumed by *D. punctatus* during larval stage significantly affected pupal body weight. Male body weights were significantly influenced by pine species and needle age. By contrast, female body weights were significantly influenced by pine species, but not by needle age. Percentages of adult emergence were also influenced either by host species or by needle age. However, there was no effect of larval food on the percentage of female progeny of the emerging adults. We found that the growth index of females was higher than that of males, and host species and needle age also had a significant impact on male growth index and female growth index.

**Table 1:** Larval and pupal life history traits (mean ± SE) of *Dendrolimus punctatus* when larvae reared on different aged host species based on univariate procedure of GLM

Parameters	Host species			Needle age		
	*P. massoniana*	*P. elliottii*	*F*	Previous-year needles	Current-year needles	*F*
Larval survivorship (%)	46.00 ± 3.93	19.67 ± 3.98	35.06**	39.00 ± 6.26	26.67 ± 6.81	7.69*
Pupal survivorship (%)	86.42 ± 5.33	41.07 ± 4.03	65.79**	70.70 ± 10.87	56.79 ± 10.63	6.20*
Larval development times (days)	32.83 ± 0.48	43.48 ± 0.60	212.27**	34.17 ± 0.69	38.73 ± 0.68	44.30**
Pupal development times (days)	7.16 ± 0.09	8.39 ± 0.22	29.62**	7.15 ± 0.12	7.76 ± 0.15	8.11**
Female pupal weight (g)	0.99 ± 0.03	0.69 ± 0.03	36.11**	0.84 ± 0.04	0.80 ± 0.06	2.73
Male pupal weight (g)	0.66 ± 0.01	0.43 ± 0.01	193.89**	0.58 ± 0.02	0.51 ± 0.02	15.05**
Percentage of adult emergence (%)	39.67 ± 4.11	8.67 ± 2.29	151.74**	30.33 ± 8.11	18.00 ± 6.11	24.02**
Percentage of female progeny (%)	40.21 ± 2.63	28.06 ± 7.10	2.18	36.93 ± 3.85	31.34 ± 7.36	0.46
Female growth index (mg/day)	31.25 ± 1.75	16.04 ± 0.76	62.04**	23.90 ± 1.84	20.15 ± 2.12	10.11**
Male growth index (mg/day)	21.06 ± 0.89	9.74 ± 0.34	130.49**	17.39 ± 1.09	13.20 ± 1.05	15.37**

Asterisk indicate significant differences between host species and needle age within parameters of larval and pupal life-history traits (*P < 0.05; **P < 0.01)

### Adult mating behavior

Very few adults originating from current-year needles of *P. elliottii* (typically fewer than 5%) emerged and males always emerged prior to females in this trial. Therefore, the mating behavior was recorded for only the adults originating from current-year needles and previous-year needles of *P. massoniana*, and previous-year needles of *P. elliottii*. The proportion of adult mating success ranged from 68.75 to 80%, and had not significantly difference among different host species (χ^2^ = 0.44, *P* = 0.51) and needle age (χ^2^ = 0.06, *P* = 0.81). Mating latency and mating duration were significantly affected by host species, but not by needle age (Table 2).

**Table 2:** Adult mating behavior and life history traits (mean ± SE) of *Dendrolimus punctatus* when larvae fed on different host species and different needle age based on independent-samples T test

Parameters	Host species			Needle age		
	*P. massoniana*	*P. elliottii*	*t*	Previous-year needles	Current-year needles	*t*
Mating latency (h)	1.37 ± 0.18	2.11 ± 0.20	− 2.72**	1.37 ± 0.18	1.59 ± 0.29	− 0.65
Mating duration (h)	16.92 ± 0.45	15.50 ± 0.28	2.68**	16.92 ± 0.45	16.38 ± 0.45	0.75
Oviposition duration (days)	5.08 ± 0.26	5.50 ± 0.27	− 1.11	5.08 ± 0.26	5.00 ± 0.54	0.16
Produced fecundity (eggs/female)	435.46 ± 19.67	202.55 ± 12.78	9.74**	435.46 ± 19.67	350.08 ± 25.88	2.56*
Remnant fecundity (eggs/female)	4.46 ± 1.49	17.46 ± 4.47	− 2.86**	4.46 ± 1.49	11.25 ± 4.16	− 1.89
Fertility (%)	76.93 ± 4.18	65.58 ± 4.64	1.77	76.93 ± 4.18	70.60 ± 7.94	0.71
Male longevity (days)	6.50 ± 0.23	9.46 ± 0.36	− 7.10**	6.50 ± 0.23	8.58 ± 0.43	− 4.72**
Female longevity (days)	6.46 ± 0.29	8.82 ± 0.27	− 5.88**	6.46 ± 0.29	7.42 ± 0.68	− 1.52
Fitness index	53.66 ± 2.42	3.57 ± 0.23	19.70**	53.66 ± 2.42	23.80 ± 1.76	8.15**

Asterisk indicate significant differences between host species and needle age within parameters of adult life history traits (*P < 0.05; **P < 0.01)

### Adult reproductive fitness

Parameters related to reproduction are presented in Table 2. No significant difference was found in the length of the oviposition period. Females laid fewer eggs and remained more eggs in their abdomen when their larvae were reared on *P. elliottii* or current-year needles than when reared on *P. massoniana* or previous-year needles. The statistics showed that the difference of produced fecundity was strongly affected by both host species and needle age, while the difference of remnant fecundity was affected by host species, but not by needle age. Females originating from *P. massoniana* or previous-year needles exhibited a higher egg hatching success than females originating from *P. elliottii* or current-year needles. However, the effect of host species and needle age on fertility was not significant. In addition, male and female longevity varied significantly among host plants. The host species had a significant influence on male longevity and female longevity, while needle age had a significant influence on male longevity but not female longevity. The values of fitness index were found to be highest for females reared on *P. massoniana* or previous-year needles than females reared on *P. elliottii* or current-year needles and produced statically difference both in host species and in needle age.

## Discussion

In traditional studies of plant–herbivore interaction, the role of host plants has attracted much attention. The direct effects of host plants to herbivorous insect are usually measured larval feeding performance in terms of survival, developmental duration, growth index and body mass, and adult reproductive fitness in terms of fecundity, fertility, longevity and fitness index [4, 5]. A growing body of studies using many Lepidoptera as research materials demonstrated that larval host species and/or cultivars significantly influenced larval performance and adult reproductive life-history traits. Individuals using the best food source as a larval host showed direct benefits in larval preference and adult reproductive performance [4–6, 11, 15, 16]. Meanwhile, nutritional quality of plants is generally considered to vary as the growing season progresses [18, 19] and the abundance and diversity of insect herbivores usually changes as their host plants age [22]. Several studies of host preference of herbivores have demonstrated that some herbivores prefer to feed on newly burst foliage [24], or prefer to feed exclusively on mature foliage [35], even food mixing [13].

In our study, we observed higher survivorship, faster development, heavier body weight and greater growth index when larvae fed with previous-year needles of *P. massoniana* than other three food sources, suggesting the larval developmental characteristics of *D. punctatus* were more strongly influenced either by larval host species or by needle age and previous-year needles of *P. massoniana* is of higher nutritional value for larvae of *D. punctatus*. These findings confirm those of an earlier study examining pine caterpillar growth and development among the same four types of foods [31]. In our observation, the development time during larval and pupal stages were significantly prolonged and obviously more insects died in these stages when larvae fed with current-year needles than larvae fed with previous-year needles. A similar tendency was observed for feeding with *P. elliottii* than *P. massoniana.* These results similar as predicted by the slow-growth–high-mortality hypothesis [6]. In addition, a prolonged development time could be a potentially important component of fitness in nature because it may increase larval exposure to predators and parasites [36–38]. The larvae reared on previous-year needles of *P. massoniana* produced heavier pupae than other three treatments. The result indicated that high quality food ingested by larvae promotes higher adult body weight [9]. We found that pine species significantly influenced male and female body weights, while needle age only significantly influenced male body weights but not female body weights, suggesting host species is the main differentiation driver. Meanwhile, females of *D. punctatus* were often heavier and larger than males. This phenomenon is common in many Lepidoptera and it has been mainly attributed to the storage of nutrients used for egg production [4, 17]. Sex ratio of emerging of adults could also determine whether the population can adapt to a certain host plant or not. Many larvae of Lepidoptera which reared on high quality hosts produced significantly higher percentage of female progeny [6]. In the present study, interestingly, although many parameters varied among host species and needle age, female progeny had similar proportion indicating no fitness penalty to speed up the larval development. *P. elliottii* and current-year needles had much reduced growth index than *P. massoniana* and previous-year needles, suggesting host species and needle age is the important factors to affect insect feed suitability. Moreover, we found that the growth index of females was higher than that of males. It is apparent that the quality of the host plant is more important to females than males in capital breeding species [14].

In our observation, the males of *D. punctatus* always emerged before females. Similar result was also observed in a study by Zhou et al. [39], in which they reported that the males always emerged approximately 2 h before females. This phenomenon of protandry is common in several other Lepidoptera [4, 9, 40]. It presents at least two advantages, by maximizing copulation opportunities for a male [41], and minimizing the pre-reproductive period of females so they emerge when most males are available [42]. However, the percentage of adult emergence difference varied according to the host species and needle age. The mating behavior of phytophagous insects is often influenced by their host plants, although this aspect is often ignored in studies of plant–herbivore interactions [4, 43]. Evaluating the mating success of adult provides insight into the effect of host plant on the reproductive success of individuals [4, 9, 44]. Different from other previous reported Lepidoptera [17], our results showed that no direct effect of larval food on adult mating success was observed. However, a strong increase in mating latency and decrease in mating duration occurred for females and males that emerged from previous-year needles of *P. massoniana*. Further analysis showed that these parameters were significantly affected by larval host species, but not by needle age, suggesting host species is the critical factor to affect mating activity of this caterpillar. The differences in the time of mating latency and mating duration of adult *D. punctatus* reared on different larval host species may be related to the quality and/or quantity of the sex pheromone that their obtained through larval feeding. Mating activity (or pheromone production) may be affected by the fact that adults have less reserves, less lipid content, etc. in a poor diet. Chemicals from host plants often synergize or otherwise enhance insect responses to sex pheromones. By these means, host plants may be used by insects to regulate or mediate sexual communication [43].

Besides larval performance, host plant species and needle age can also influence the reproductive output of herbivorous insects. Previous theoretical and experimental evidence demonstrated that larval food strongly influenced female fecundity (via pupal weight and the duration of egg laying), fertility, as well as their longevity [2, 4, 6, 11, 15, 17]. Form our study, we found that females emerged from larvae reared on *P. elliottii* and current-year needles laid significantly fewer eggs, remained more eggs in their abdomen and lived longer than females from larvae reared on *P. massoniana* and previous-year needles. Previous studies suggested that fecundity is generally tightly coupled to body size of females in capital breeding insects [45, 46]. Although the potential relationships between female body weight and fecundity have not been well compared, our results support the hypothesis that a high-quality food ingested by larvae promoting higher pupal body weight enhances female fecundity [45, 46]. In addition, the differences in fecundity on different host species and needle age also demonstrated that the chemical composition of the host plants play an important role as oviposition stimulant for Lepidopterans [6, 47, 48]. However, we found no significant differences in oviposition duration and fertility. This may suggest that adult can suffer the difference of food quality and transfer the same nutrients used for egg hatching. Through overall analysis, we found that females originating from *P. massoniana* and previous-year needles exhibited the highest fitness index than females originating from *P. elliottii* and current-year needles, further suggesting *P. massoniana* and previous-year needles contributed the best to the reproductive fitness of *D. punctatus*. These findings are consistent with the results of previous study in which old needles generally have lower water content, higher in fiber, soluble carbohydrate and protein and it is helpful to the development and reproduction of *D. punctatus* [29, 49].

In summary, all the parameters measured in our experiments clearly indicated that the studied populations of *D. punctatus* physiologically best adapted to use old needles of masson pine *P. massoniana* as a host plant. Same situation also occurs in the field [29, 49]. So, the quality of host plants as larval food should be taken into account as a factor affecting the population dynamics of *D. punctatus*. Conversely, slash pine *P. elliottii* is not suitable for survival of larvae and reproduction of adults, indicating this pine species may produce higher anti-herbivore defensive substance than masson pine *P. massoniana*. This paper provides basic knowledge for further study of the plant anti-herbivore mechanism and the development of plant defense-based pest control strategies of this species. In this study, however, we have only compared two host species with two aged class. Indeed, *D. punctatus* has been reported to cause damage to other pine trees such as loblolly pine *P. taeda*, red pine *P. densiflora*, and black pine *P. thunbergii* [26, 29], although these host plants are not widely distributed in the south of China. For further understanding of the relationships between the host preference and reproductive capability of *D. punctatus*, it is necessary to compare the development and reproductive performances on different host plants. In addition, we have only considered the direct effects of the host plant by affecting larval performance, adult mating behavior and reproductive fitness in this study. To fully understand plant–herbivore interactions, further studies should also be conducted to investigate indirect effects of host plants like its susceptibility to natural enemies of the herbivores, such as predators, parasitoids and pathogens [1, 4, 50].

## Conclusion

Results from our present study indicate that larval developmental traits and adult reproductive fitness were significantly influenced both by plant species and by needle age used as larval food sources in *D. punctatus*. Overall, individuals using *P. massoniana* and previous-year needles as a larval host showed a significant decrease in developmental duration, a significant increase in survival, body size, and growth index compared with those using *P. elliottii* and current-year needles as larval host. In addition, adults derived from *P. massoniana* and previous-year needles exhibited a higher mating success, a shorter mating latency and a longer mating duration. Meanwhile, this caterpillar acquired more fitness benefits in terms of higher fecundity, fertility and fitness index. These results reveal the importance of larval host plants on larval performance and adult reproductive fitness in this capital breeding insect and provides strong evidence that previous-year needles of *P. massoniana* is the best host for *D. punctatus*.
